# Mulibrey Nanism: Clinical Spectrum and Molecular Pathogenesis

**DOI:** 10.3390/ijms27094074

**Published:** 2026-05-01

**Authors:** Hubert Piwar, Jan Pawlasek, Michal Ordak

**Affiliations:** 1 Department of Pharmacotherapy and Pharmaceutical Care, Faculty of Pharmacy, Medical University of Warsaw, Banacha 1 Str., 02-097 Warsaw, Poland; 2 Centre of Regenerative Medicine, Medical University of Bialystok, Waszyngtona 15B Str., 15-269 Bialystok, Poland

**Keywords:** Mulibrey nanism, TRIM37, short stature

## Abstract

Mulibrey nanism is a rare autosomal recessive multisystem disorder caused by biallelic loss of function variants in TRIM37 encoding a peroxisomal E3 ubiquitin ligase. Initially described in Finland, where it remains most prevalent due to a founder mutation, the condition is now recognized worldwide and is characterized by severe prenatal-onset growth failure, distinctive craniofacial features, radiological abnormalities, ocular findings, and hepatopathy. Although its clinical spectrum extends far beyond these core manifestations, the major determinant of morbidity and mortality is progressive cardiovascular disease, including constrictive pericarditis and restrictive cardiomyopathy. Additional features include metabolic dysfunction such as insulin resistance and type 2 diabetes, gonadal insufficiency, skeletal abnormalities including fibrous dysplasia, and an increased risk of benign and malignant tumours. The clinical course evolves across the lifespan from early growth and developmental abnormalities to progressive multisystem disease in adolescence and adulthood. Recent advances have expanded understanding of TRIM37 function, linking it to mTORC1 TFEB signalling autophagy, centrosome integrity, extracellular matrix regulation, and immune cell function, providing mechanistic insights into tumour predisposition, skeletal pathology, and immune dysregulation. Management remains supportive and requires multidisciplinary care with emphasis on early recognition and treatment of cardiac disease, metabolic complications, and malignancy risk. Prognosis is variable but improves with early diagnosis and appropriate surveillance. This review summarises the clinical spectrum molecular mechanisms and current management of Mulibrey nanism and highlights priorities for future research.

## 1. Introduction

Mulibrey nanism (MUL; muscle-liver-brain-eye; OMIM #253250) is an autosomal recessive multisystem disorder first described in the early 1970s and now recognized as a distinct prenatal onset growth disorder [1,2]. It is characterized by severe prenatal growth failure, characteristic craniofacial features (triangular face, frontal bossing, scaphocephaly), muscular hypotonia, specific radiological findings, ocular findings including yellowish dots in the fundi, and hepatomegaly [2]. The clinical spectrum of disease extends well beyond the MUL acronym to include progressive cardiovascular disease, particularly combined pericardial and myocardial disease, including constrictive pericarditis and restrictive cardiomyopathy, which is the leading cause of morbidity and mortality [2,3,4], as well as endocrine and metabolic dysfunction, including severe insulin resistance, metabolic syndrome and type 2 diabetes [2,5]. Gonadal dysfunction, including impaired sexual maturation and reduced fertility, has also been reported in affected patients [2,6,7]. In addition, patients with MUL develop skeletal complications including fibrous dysplasia and increased fracture risk [8], as well as a predisposition to benign and malignant tumours, including Wilms tumor and other neoplasms [9,10]. At the molecular level, MUL is caused by biallelic loss-of-function variants in TRIM37, which encodes a tripartite motif E3 ubiquitin ligase with peroxisomal localization [11,12]. Recent studies have broadened our understanding of TRIM37 function, linking the protein to mTORC1–TFEB signalling and autophagy [13], centrosome integrity and mitotic fidelity [14,15], ECM regulation in chondrocytes [16] which may contribute to skeletal pathology [8], and immune cell function [17,18]. In this review, we discuss the clinicopathologic spectrum of MUL, summarize recent advances in the biology of TRIM37, and highlight priorities for future diagnosis and surveillance. A comprehensive literature search was performed using major biomedical databases, including PubMed, Scopus, ScienceDirect, and Google Scholar, to identify relevant publications on Mulibrey nanism and TRIM37. The analysis included clinical, genetic, and molecular data reported in case series and cohort studies, with particular focus on phenotypic features, disease progression, and underlying mechanisms. Data from published reports were reviewed and integrated.

## 2. Epidemiology and Natural History

### 2.1. Epidemiology

Worldwide, MUL remains extremely rare, but occurs predominantly in Finland due to a founder effect related to the “Fin-major” TRIM37 mutation [8,11,19]. Early reports described only a few individuals. Subsequent reports show gradual case accumulation rather than fixed occurrence rates.

### 2.2. Documented Case Numbers over Time

The number of reported cases has grown steadily as awareness of the disorder has expanded globally (Table 1). Table 1 summarises the cumulative counts reported in key publications.

### 2.3. Incidence Estimates

Global population rates are generally not calculated in MUL studies. Instead, authors report numbers of diagnosed, documented, or genetically verified individuals, which reflect both the year of publication and the thoroughness of case detection. In older Finnish sources, the incidence of MUL in Finland has been estimated at approximately 1 in 40,000 births [22] with a later report citing 1 in 37,000 births in the same population [19].

### 2.4. Geographic Distribution

Although the majority of patients are Finnish, MUL has been documented across multiple ethnic backgrounds. Case reports from Spain, Argentina, Turkey, Iran, and Syria confirm that MUL occurs well beyond the Finnish population [23,24,25,26].

### 2.5. Natural History: Clinical Phases

The clinical burden of MUL evolves across life stages, with distinct manifestations predominating at each phase:

Prenatal and infancy: Intrauterine growth restriction with postnatal failure to thrive and characteristic dysmorphology. Feeding difficulties and recurrent respiratory infections are common [2,20].

Childhood: Progressive cardiac and multiorgan manifestations. Diastolic dysfunction and restrictive-constrictive physiology may require evaluation beyond routine echocardiography when clinical suspicion persists [3,27,28].

Adolescence and adulthood: Long-term follow-up is increasingly dominated by heart failure, hepatopathy, metabolic disease, and gonadal dysfunction with infertility, while tumour risk remains important across the lifespan and includes both childhood and adult neoplasms [3,5,7,9,29].

## 3. Genetics and Molecular Pathogenesis

### 3.1. Gene Discovery and Identification of TRIM37

In Finnish families, linkage and linkage disequilibrium mapping first assigned MUL to chromosome 17q, narrowing the critical region and enabling positional cloning [22]. A high-resolution physical and genetic mapping study then built a bacterial clone contig across the overlapping Meckel syndrome and MUL critical regions, providing the markers and transcript map needed for gene identification [30]. Finally, TRIM37 was identified as the causative gene: multiple independent pathogenic variants (including frameshift, splice-site, and insertion/deletion variants) were shown to predict truncated protein products, establishing MUL as a monogenic disorder caused by a broadly expressed member of the TRIM/RBCC protein family [31]. Intragenic rearrangements and complete gene deletions have also been reported, likely promoted by the abundant Alu repetitive elements in this genomic region [21]. A Trim37-deficient mouse line recapitulates several features of the human disease, including fatty liver/hepatic changes and cardiomyopathy, supporting loss of function as the primary pathogenetic mechanism [32].

### 3.2. TRIM37 and Peroxisomal Function

TRIM37 was first described as a peroxisomal RBCC (RING-B-box-coiled-coil) protein, which led to the initial classification of MUL as a peroxisomal disorder. Early studies showed that patient fibroblasts lacked detectable TRIM37 protein while peroxisome number and morphology appeared normal, suggesting a TRIM37-specific defect rather than a global failure of peroxisome biogenesis [11]. Subsequent work in human cells proposed a mechanistic link, showing that TRIM37 ubiquitylates and stabilises the peroxisomal import receptor PEX5, thereby promoting peroxisomal matrix protein import. Notably, TRIM37-depleted human cells exhibited impaired PEX5 stability and peroxisomal protein import, a finding that contrasted with earlier observations and that the authors attributed to variable residual TRIM37 expression among patient cell lines carrying the same splice-site mutation [33]. It should be noted, however, that Trim37 knockout mice and mouse cell lines depleted of TRIM37 do not exhibit a peroxisomal phenotype [33] suggesting species-specific differences in TRIM37-dependent peroxisomal import that complicate extrapolation from mouse models to human disease.

### 3.3. TRIM37 and mTORC1–TFEB Signalling

TRIM37 has also been linked to the regulation of mTORC1 signalling. In human cells, TRIM37 interacts with MTOR and RRAGB, enhances the MTOR–RRAGB interaction and promotes lysosomal localisation of MTOR, thereby supporting amino acid-stimulated mTORC1 activation. Loss of TRIM37 inhibits mTORC1 signalling and reduces TFEB phosphorylation, resulting in TFEB nuclear translocation and transcriptional upregulation of genes involved in autophagy and lysosome biogenesis. The resulting enhanced autophagy may serve as a pro-survival mechanism in TRIM37-deficient cells, which may be relevant to tumor predisposition in MUL [13].

### 3.4. TRIM37 and Centrosomal Integrity

TRIM37 also protects mitotic fidelity at the centrosome [15,34]. Without TRIM37 centrobin can aggregate into condensates that acquire microtubule-organizing activity and form ectopic spindle poles, causing transient multipolar spindles and chromosome missegregation in both experimental models and patient-derived cells [14,15,35]. Recent studies indicate that TRIM37 counteracts this by recognising substrates via its TRAF (MATH) domain [35,36] and becoming fully active only when substrate proteins accumulate into larger assemblies, which triggers B-box-mediated oligomerisation and RING domain dimerisation—a stepwise activation process operating at the mesoscale level that enables the selective degradation of centrosomal proteins once they cluster into higher-order assemblies [36]. This mechanism provides a plausible molecular route to aneuploidy and tumour predisposition in MUL, although direct evidence at the level of human clinical causality remains limited [35,36].

### 3.5. TRIM37 Deficiency and Skeletal Pathology

Experimental evidence links TRIM37 deficiency to skeletal pathology. Brigant et al. (2020) demonstrated that TRIM37 is highly expressed during mitosis in CHON-002 chondrocytes and is post-transcriptionally regulated by miR-223, suggesting a role in chondrocyte proliferation [37]. In a subsequent study, Brigant et al. (2024) showed that downregulation of TRIM37 in a chondrocyte model is associated with altered ECM protein expression, altered collagen levels, and changes in SPARC expression [16]. Additionally, TRIM37 loss is linked to dysregulation of ECM-organisation and TGF-β signalling pathways, both of which are critical for tissue organisation and cell proliferation. These findings suggest that TRIM37 deficiency may alter ECM protein expression in chondrocytes, which could contribute to impaired longitudinal bone growth. However, the cell line used (CHON-002) is negative for type II collagen and findings were obtained at a single time point, so extrapolation to growth plate physiology in vivo remains speculative. Separately, MUL patients display a broader skeletal phenotype that includes fibrous dysplasia and increased fracture risk [8], the pathogenesis of which remains unclear.

### 3.6. TRIM37 and Immune Dysfunction

Immune involvement in MUL has been explored from two directions: detailed immunophenotyping of an affected patient revealed T-cell lymphopenia, hypogammaglobulinaemia and impaired T-cell proliferation, although the authors noted that these abnormalities may partly reflect PLE secondary to CHF rather than a primary immune defect [17]. A parallel experimental study provided a direct mechanistic link: in a healthy immune system, TRIM37 stabilises BCL6 via its E3 ligase activity, catalysing atypical K27/K29-linked polyubiquitination that protects BCL6 from proteasomal degradation. BCL6 is a key regulator of T follicular helper (TFH) cell differentiation and the generation of high-affinity antibodies [18]. In Trim37 knockout mice, BCL6 protein stability is directly reduced, providing direct evidence for a mechanistic link between TRIM37 loss and impaired TFH-cell differentiation. Trim37 FINmajor knock-in mice show a concordant phenotype, with markedly decreased CXCR5+Bcl6+ TFH cells, defective germinal centre responses, weakened humoral immunity, and increased susceptibility to influenza infection [18].

### 3.7. Genotype–Phenotype Correlations

Clinical genotype–phenotype correlations in MUL remain difficult to establish, in part because most patients carry truncating mutations and cohorts are small. Phenotypic variability has been observed even between siblings with the same TRIM37 genotype [2]. At the molecular level, however, recent studies have revealed domain-specific functional consequences: the G322V missense variant in the TRAF (also known as MATH) domain disrupts both substrate engagement at centrosomal assemblies [36] and BCL6 ubiquitination required for TFH-cell differentiation [18], while the C109S B-box variant impairs oligomerization needed for mesoscale E3 ligase activation [36]. These findings indicate that the position and nature of a TRIM37 mutation may selectively compromise distinct molecular functions, although whether this translates into recognisable clinical differences among patients remains an open question. Outside founder populations, the genetic landscape is more diverse and can include complex structural and splice-altering variants. Mozzillo et al. described a patient carrying a large maternal 17q22 chromosomal deletion (encompassing TRIM37 and SKA2) together with a paternal intronic splice-site variant (c.1949-12A>G). mRNA analysis confirmed aberrant splicing resulting in a frameshift and premature termination of translation [38]. Most recently, Zodanu et al. (2026) identified two novel heterozygous missense variants—c.199C>T (p.Arg67Cys) in the RING–B-box linker region and c.2041C>G (p.Gln681Glu) in the C-terminal region—in a preterm infant with an unusually severe and atypical phenotype that included complex structural congenital heart disease (interrupted aortic arch type B, ventricular septal defect, and persistent left superior vena cava), omphalocele, cleft palate, and multicystic dysplastic kidney [39]. Both variants were classified as variants of uncertain significance. Trio sequencing was not available to confirm compound heterozygosity, and the marked phenotypic divergence from typical MUL raises the possibility that additional genetic or environmental factors contributed to the clinical presentation. Table 2 presents published TRIM37 mutations associated with MUL.

## 4. Clinical Presentation

### 4.1. Overview and Diagnostic Context

Clinically, MUL is characterized by severe prenatal-onset growth failure, a distinctive craniofacial and radiological phenotype, and multisystem involvement that often becomes more evident with age. Prognosis is determined primarily by combined pericardial and myocardial disease, which may progress to CHF [2,3].

### 4.2. Clinical Diagnostic Criteria

In the Finnish national cohort described by Karlberg et al. in their 2004 diagnostic study (85 patients), a diagnostic framework was proposed: diagnosis requires either three major signs and one minor sign, or two major signs and three minor signs. Major signs comprise (i) growth failure (small for gestational age with absent catch-up growth, or height ≤ −2.5 SDS in childhood/≤−3.0 SDS in adults), (ii) characteristic radiology (slender long bones with thick cortices and narrow medullary canals, and/or a low shallow J-shaped sella turcica), (iii) characteristic craniofacial features (scaphocephaly, triangular face, high/broad forehead, low nasal bridge, telecanthus), (iv) characteristic ocular findings (yellowish retinal dots in the mid-peripheral fundus), and (v) MUL in a sibling. Minor signs comprise a peculiar high-pitched voice, hepatomegaly, cutaneous naevi flammei, and fibrous dysplasia of long bones [2]. Earlier reports identified short stature, a J-shaped sella turcica, yellowish retinal fundus dots, and pericardial constriction as particularly informative recognition features [1], while non-Finnish case reports also noted phenotypic variability outside founder populations [24].

### 4.3. Growth Pattern and General Habitus

Growth failure begins prenatally. In the Finnish diagnostic cohort of Karlberg et al. (2004), 95% of patients showed prenatal-onset growth restriction without postnatal catch-up growth, and the median age at diagnosis was approximately 2 years [2]. Feeding difficulties and recurrent respiratory tract infections are common in infancy. Affected individuals typically have a gracile habitus, with thin extremities, relatively short proximal limb segments, narrow shoulders, and relative macrocephaly. Common craniofacial features include a triangular face, a broad and high forehead, a low nasal bridge, and scaphocephaly with occipitofrontal bossing. Longitudinal growth analysis by Karlberg et al. in Pediatrics (2007) examined 72 patients and showed continuous deceleration in height and weight-for-height during infancy, followed by partial catch-up by school age. Final adult height averaged approximately 136 cm in women and 150 cm in men [20]. In the more recent skeletal cohort of Karlberg et al. (2025), in which 85% of 33 patients had received GH therapy, median adult height was 152 cm in males and 142 cm in females [8]. Body composition changes markedly with age: while prepubertal children are characteristically slim, clinically evident abdominal obesity develops after puberty, with 42% of adults being overweight in the metabolic cohort of Karlberg et al. (2005) [5].

### 4.4. Craniofacial, Dental and Ophthalmologic Phenotype

In the Finnish series reported by Karlberg et al. (2004), characteristic craniofacial features were present in approximately 90% of patients at diagnosis and included scaphocephaly, triangular face, a high and broad forehead, a low nasal bridge, and telecanthus [2]. Dental crowding was present in 50% of the Finnish cohort. A peculiar high-pitched voice is another frequent diagnostic clue, reported in 96% of the Finnish cohort described by Karlberg et al. (2004). Ocular abnormalities are highly characteristic and contribute to diagnosis: yellowish dots in the mid-peripheral retina were observed in 79–80% of Finnish patients, often together with additional pigmentary changes including retinal hypopigmentation and pigment dispersion [2].

### 4.5. Musculoskeletal and Radiological Phenotype

Radiological hallmarks include slender long bones with thick cortices and narrow medullary canals (93%) and a low, shallow J-shaped sella turcica (89%). The thorax is often small and bell-shaped with thin ribs, contributing to the characteristic somatic proportions. The more detailed radiographic analysis by Karlberg et al. (2025) additionally documented bowing of the long bones in all 33 patients examined, broad metaphyses suggesting overmodelling, and tall vertebral bodies, most prominent in adult patients but apparent already in childhood [8]. These findings led the authors to propose MUL as a slender bone dysplasia [8]. Fibrous dysplasia of the long bones was recorded in 25% of the Finnish diagnostic cohort [2]. More recent radiographic assessment of 33 patients found a substantially higher prevalence of 58%, with 52% having a history of fractures, establishing fibrous dysplasia as an important contributor to skeletal morbidity.

### 4.6. Cardiovascular Phenotype: Pericardial and Myocardial Disease

Cardiac involvement is the principal determinant of outcome. MUL-associated heart disease typically combines constrictive pericarditis—often accompanied by pericardial thickening or calcification—with myocardial involvement, including hypertrophy and variable but mostly mild fibrosis, thereby producing a restrictive—constrictive filling physiology, often with preserved ejection fraction [3]. However, the prospective echocardiographic study by Sarkola et al. (2022) of 23 paediatric patients demonstrated that tissue Doppler and speckle tracking strain imaging can reveal mild biventricular systolic dysfunction even when conventional ejection fraction is preserved, and that pericardial constriction may be haemodynamically significant despite a normal-appearing pericardium on echocardiography [4]. In the Circulation series by Lipsanen-Nyman et al. (2003), which followed 49 Finnish patients for up to 25 years, 51% developed CHF, 39% underwent pericardiectomy for constrictive pericarditis, and 22% died of cardiac causes. Although pericardiectomy generally provides clinical benefit, heart failure may recur or persist because of concomitant myocardial disease [3]. Recent paediatric case literature, including the report by Kreinbrook et al. (2024), suggests that echocardiography may underestimate restrictive cardiomyopathy, making invasive hemodynamic assessment advisable in selected patients [28]. Arrhythmias, including atrial fibrillation, may develop in the context of advanced cardiac disease [3].

### 4.7. Hepatic and Gastrointestinal Manifestations

Hepatomegaly is common (45% at diagnosis in the Finnish diagnostic cohort of Karlberg et al., 2004) and may reflect congestive hepatopathy related to cardiac disease and/or focal lesions [2]. Later studies have also identified intrinsic hepatic pathology including steatosis and fibrosis [47]. Mild transaminase elevation was frequent, whereas routine laboratory abnormalities were otherwise often non-specific [2]. In the national Liver International study by Sivunen et al. (2022), which included data from biopsies, pre-pericardiectomy cohorts, outpatient assessments, and autopsy material, chronic liver involvement included sinusoidal dilatation/congestion, steatosis, and fibrosis, with progression to cirrhosis particularly after the age of 40 [47]. Notably, conventional liver biochemistry was relatively insensitive in detecting these changes, suggesting that current screening tools may underestimate the extent of hepatic involvement. The authors noted that an intrinsic TRIM37-dependent contribution to liver pathology, independent of cardiac disease, cannot be excluded. Protein-losing enteropathy has been described in the setting of severe right-sided heart failure and may contribute to oedema, hypoalbuminaemia, and in some cases secondary immune dysfunction [17,48].

### 4.8. Metabolic and Endocrine Phenotype

Metabolic complications become more pronounced with age. In the metabolic study by Karlberg et al. (2005), which included 65 patients aged 1.1–55 years, insulin resistance and dyslipidaemia increased markedly over time: approximately half of adults had type 2 diabetes, 42% had impaired glucose tolerance, and 70% met criteria for metabolic syndrome [5]. Insulin resistance and fatty liver were already detectable in many slim prepubertal children. Gonadal dysfunction is another important component of the phenotype. Premature ovarian failure was first described by Karlberg, Tiitinen, and Lipsanen-Nyman (2004) [6], with later studies documenting that ovarian follicle depletion begins in infancy, leading to premature ovarian insufficiency [29], while primary testicular failure and infertility were reported in males [7].

### 4.9. Renal and Urinary Tract Manifestations

Renal and urinary tract anomalies, renal tumours, and cystic lesions have all been reported. In the retrospective renal study by Sivunen et al. (Pediatric Nephrology, 2017) of 101 Finnish patients, structural kidney/urinary anomalies were identified in 13%, renal tumours in 14%, and macroscopic cystic lesions in 43% of the 81 patients examined by ultrasound [51]. Histology commonly demonstrated glomerular cysts (present in 87% of biopsied samples), prominent thick-walled vessels, and moderate upregulation of EMT markers (α-SMA, vimentin), although fibrosis markers (collagen I, CTGF) did not differ significantly from controls. Although renal function was preserved in most patients, hypertension (25%) and mildly decreased GFR (23%) were observed in a subset [51].

### 4.10. Respiratory Phenotype

Respiratory infections are common early in life [2]. In the pulmonary function study by Sivunen et al. (Pediatric Pulmonology, 2020) of 33 Finnish patients, a typical restrictive pattern was observed, with markedly reduced lung volumes (TLC and FVC approximately 51–63% predicted) but no evidence of airway obstruction [52]. Specific diffusing capacity (DLCO/VA) was significantly increased (130–148% predicted), suggesting preserved pulmonary tissue function despite reduced lung volumes. These findings suggest that asthma-like symptoms in MUL should be interpreted with caution, as only one of 32 tested patients had a positive bronchodilation test, and emphasize the contribution of body size, thoracic configuration, and cardiopulmonary interactions [52].

### 4.11. Tumour Predisposition and Neoplasia Spectrum

Both benign lesions and malignancies are common in MUL. In the national tumour study by Karlberg et al. (Journal of Pathology, 2009), tumours or tumour-like lesions were identified in 74% of 89 Finnish patients aged 0.7–76 years [10]. Malignancies occurred in 15% (15 tumours in 13 patients), affecting the kidneys (including five Wilms tumours), thyroid gland, and gynaecological organs, as well as isolated cases of gastrointestinal carcinoid, neuropituitary Langerhans cell histiocytosis, and acute lymphoblastic leukaemia, alongside a range of benign vascular and adenomatous lesions [9]. Renal tumours were particularly notable: in the larger renal cohort of Sivunen et al. (2017), 14% of 101 patients had renal neoplasms, including Wilms tumour (mean age at diagnosis 2.5 years) and papillary renal carcinoma in adults, underscoring the need for lifelong renal surveillance [51]. Thyroid papillary carcinomas were also reported in adults [9]. Gynecological tumours—particularly ovarian fibrothecomas—appear overrepresented in women with MUL and may be bilateral or multifocal. This association was analysed in detail by Karlberg et al. (2009), who also implicated TRIM37 in their pathogenesis [10]. Upasana et al. (2022) noted that treatment of renal malignancies can be feasible but requires attention to coexisting cardiac disease and other systemic complications [9,40].

### 4.12. Neurodevelopment and Other Features

Severe neurological impairment is not a typical feature of MUL, but mild hypotonia and variable delay in early developmental milestones have been described. In the Finnish diagnostic cohort reported by Karlberg et al. (2004), mild muscular hypotonicity (68%) was the only neurological finding [2]. The ages of first steps and first words were variable, and hydrocephalus was suspected in a minority of infants. Cutaneous naevi flammei are common (65% in the Finnish cohort described by Karlberg et al., 2004), most often on the lower limbs. Skeletal asymmetry and congenital anomalies, including structural cardiac, renal, and genital anomalies, have also been reported.

### 4.13. Immune Dysfunction: Emerging but Heterogeneous

Although recurrent infections are common in early childhood, overt immunodeficiency is not a universal feature of MUL. Nevertheless, hypogammaglobulinaemia and CD4+ T-cell defects have been documented in individual patients [17,50,53]. The possible underlying mechanisms, including PLE and a primary TRIM37-dependent immune defect, are discussed in the Section 3 above.

### 4.14. Differential Diagnosis and Practical Considerations

The main phenotypic overlaps among disorders with prenatal-onset growth re-striction include Silver–Russell syndrome and 3-M syndrome. In clinical practice, howev-er, hepatomegaly and combined pericardial and myocardial disease —together with the distinctive radiological and ocular signs such as a J-shaped sella turcica, slender long bones, and yellowish retinal dots—should prompt early clinical suspicion and targeted TRIM37 testing, particularly in cases of prenatal-onset growth restriction of unknown cause [2,19].

## 5. Management, Treatment and Prognosis

### 5.1. General Principles

There is currently no curative treatment for MUL. Given the multisystem nature of the disorder [2,3], management is directed at individual complications, with goals of early recognition, prevention of end-organ damage through proactive surveillance, and timely intervention, particularly for cardiac and oncological complications. Because the clinical burden evolves throughout life and varies considerably between individuals, care requires longitudinal multidisciplinary input from paediatric and adult subspecialists in cardiology, endocrinology, hepatology, oncology, orthopaedics, and ophthalmology. The following sections draw on cohort-level evidence from the Finnish national patient series, supplemented by smaller cohort studies and case series from non-Finnish populations. Where evidence is limited to case reports, this is noted explicitly [2,3,9,20].

### 5.2. Cardiovascular Management

Cardiac disease is the principal determinant of prognosis in MUL and requires close clinical attention. The pathophysiology combines constrictive pericarditis (characterised by non-inflammatory fibrous thickening of the pericardial layers, often with adhesions to the epicardium and, in advanced cases, calcific plaques) with intrinsic myocardial involvement comprising ventricular hypertrophy and variable but mostly mild fibrosis. This results in a mixed constrictive-restrictive physiology, often with preserved left ventricular ejection fraction, although mild biventricular systolic abnormalities, detectable by tissue Doppler and speckle tracking, may coexist [3,4].

### 5.3. Echocardiographic Surveillance and Cardiac Assessment

Regular echocardiographic follow-up is the cornerstone of cardiac surveillance in MUL, although interpretation requires awareness of its limitations. Importantly, pericardial thickening is frequently not detectable by standard echocardiography despite haemodynamically significant constriction: in the prospective paediatric cohort of Sarkola et al. (2022), which enrolled 23 MUL patients and 23 individually matched healthy controls, pericardial appearance was not different from that of control subjects, whereas functional markers of constriction (septal bounce, pronounced hepatic vein atrial reversal, exaggerated respiratory variation in right heart inflow-outflow velocities, and decreased inferior vena cava collapse during inspiration) were clearly present in patients who had not undergone pericardiectomy [4]. This has direct clinical implications: the decision to proceed with pericardiectomy should not rely on pericardial thickness alone, as this may appear normal even in the presence of significant constriction [4]. In patients with clinical signs of heart failure or haemodynamic compromise, invasive catheterisation demonstrating elevated and equalised atrial pressures can confirm constrictive physiology when non-invasive assessment is equivocal. Cardiac magnetic resonance imaging may provide complementary information about pericardial and myocardial involvement and should be considered in selected cases [4,28,54]. In patients who have undergone pericardiectomy, ongoing echocardiographic surveillance remains essential: myocardial motion abnormalities and persistent heart failure were documented in 3 of 6 post-pericardiectomy patients in the Sarkola cohort, consistent with progressive intrinsic myocardial disease. More broadly, decreased right ventricular free wall longitudinal systolic strain and bilateral longitudinal myocardial systolic velocities indicative of mild biventricular systolic dysfunction were common among MUL patients regardless of surgical history [4].

### 5.4. Pericardiectomy: Indications, Timing and Outcomes

Pericardiectomy is the established surgical treatment for MUL-associated constrictive pericarditis. The most comprehensive outcome data derive from the Circulation series by Lipsanen-Nyman et al. (2003), which followed 49 Finnish patients for up to 25 years [3]. Of the 19 patients who underwent pericardiectomy, 12 derived lasting clinical benefit, however, 6 died subsequently of unrelieved or recurrent CHF, and 1 died early of a noncardiac cause. The authors concluded that while pericardiectomy generally provides benefit, CHF recurs in approximately one third of patients because of coexisting myocardial involvement that pericardiectomy cannot address. Available evidence from case series supports early intervention, before advanced pericardial adherence and irreversible myocardial restriction develop [3,45,48]. Yasuhara et al. (2018) described the first Japanese series [45]. Total pericardiectomy was successful in one patient who remained asymptomatic at five-year follow-up. Another patient died of heart failure before surgery could be performed. In a third patient, partial pericardiectomy initially improved cardiac function, but the child subsequently died of multiple organ failure due to fungal pneumonia, precluding conclusions about the adequacy of incomplete resection. The authors suggested that early diagnosis and total pericardiectomy before pericardial adhesion develops may improve outcomes, although this remains to be confirmed in larger series. The case reported by Kumpf et al. (2013) illustrates the hazard of delayed surgery: a 12-year-old child with a novel TRIM37 mutation developed refractory CHF following late pericardiectomy and died two months postoperatively [48]. By contrast, Cordova Sanchez et al. (2022) described a patient who underwent pericardiectomy at age 12 and subsequently maintained stable mild diastolic dysfunction for approximately 44 years, living a relatively normal life, although her heart failure ultimately deteriorated in her final year, leading to death—illustrating both the potential for prolonged benefit from early surgery and the progressive nature of MUL cardiac disease [55]. The available case reports suggest that total pericardiectomy may offer more complete relief than partial resection: in the Yasuhara series [45], total pericardiectomy led to sustained improvement, whereas partial resection in a sibling was followed by a fatal outcome, although the contribution of other factors cannot be excluded given the small number of cases. When pericardial layers are densely adherent to the epicardium, a complication more common in delayed presentations, resection becomes technically more hazardous and the likelihood of complete relief is reduced [45]. Minimally invasive thoracoscopic pericardiectomy has been reported in a single case with good short-term palliation, though long-term follow-up is lacking [56].

### 5.5. Heart Transplantation

When end-stage heart failure persists or progresses despite maximal medical therapy, particularly in the presence of combined pericardial and myocardial disease, heart transplantation may be considered as a definitive treatment. Anwer et al. (2020) reported successful transplantation in a patient with MUL and extensive calcification penetrating the myocardium, proposing that transplantation should be considered in appropriately selected end-stage cases [57]. Evidence is limited to individual case reports, and eligibility requires careful assessment given the multisystem disease burden (including hepatic cirrhosis, metabolic syndrome, and potential malignancy) [28,57].

### 5.6. Metabolic and Endocrine Management

Metabolic complications in MUL are progressive and, if untreated, contribute to long-term morbidity. In the metabolic study by Karlberg et al. (2005) of 65 patients aged 1.1–55 years, 70% of patients aged 20 years and over met criteria for metabolic syndrome, approximately half had type 2 diabetes mellitus, and 42% had impaired glucose tolerance [5]. Notably, evidence of insulin resistance and hepatic steatosis was already detectable in prepubertal children, indicating that metabolic abnormalities can be present from early childhood. These findings support regular metabolic surveillance, including assessment of glucose metabolism, lipid profile, and blood pressure.

### 5.7. Growth Hormone Therapy

Growth hormone (GH) therapy has been used in MUL to improve growth. In the longitudinal growth study by Karlberg et al. (2007), which examined 72 patients, long-term GH treatment was associated with an improvement in prepubertal growth velocity [20]. However, the gain in final adult height was modest, estimated at less than 5 cm above the untreated trajectory (approximately 0.6 height SDS). GH-treated adults were slimmer and showed favourable metabolic profiles compared with untreated adults, suggesting that GH therapy in MUL was safe and did not aggravate the disorder’s baseline insulin resistance. The authors note that among children with severe co-morbidities, specifically CHF or prolonged severe feeding difficulties, GH response was very poor and treatment was discontinued in three cases after less than 2.5 years, suggesting that severe co-morbidities, including CHF and prolonged feeding difficulties, may limit treatment response.

### 5.8. Gonadal Insufficiency and Fertility

Sexual maturation failure and hypergonadotropic hypogonadism are recognised features of MUL in both sexes and were first described by Karlberg, Tiitinen, and Lipsanen-Nyman in 2004 [6]. In women, the detailed cohort study by Karlberg et al. (2018) documented progressive premature ovarian insufficiency: in the cross-sectional evaluation of 19 pubertal or postpubertal patients, none had normal ovarian morphology, 82% had no or fewer than two visible antral follicles on ultrasonography, and anti-Müllerian hormone was undetectable in 89% [29]. Although the median age at menarche was 12.5 years, close to the population average, regular menstruation 10 years after menarche was observed in only 8% of women. Given the progressive loss of ovarian reserve, fertility preservation counselling may be considered early, before the loss becomes clinically apparent. Hormone replacement therapy may be appropriate for symptomatic hypogonadism and for reducing the long-term risks of oestrogen deficiency, following general principles of premature ovarian insufficiency management. In men, the cohort study by Karlberg et al. (2011) of 28 patients found that all adult males had primary testicular failure, with hypergonadotropic hypogonadism with elevated FSH and LH, low inhibin B, small testicular volume, and severe oligoasthenozoospermia or azoospermia in all semen samples [7]. Puberty initiated spontaneously in all patients (median age 12.6 years), but testicular growth proceeded slowly after midpuberty. Masculinisation, although slow, was usually satisfactory but remained incomplete in the majority (72% did not reach Tanner stage G5P5). Fertility is severely compromised, although assisted reproduction was successful in one reported case, suggesting early specialist referral to discuss options including sperm extraction. Among men in stable partnerships, the majority reported satisfactory sexual function, although about a third of adult men in the cohort had no sexual relationships. Testosterone replacement therapy may be considered in patients with symptomatic androgen deficiency.

### 5.9. Hepatic Management

Liver disease in MUL has two components: congestive hepatopathy from chronic right-sided heart failure, and intrinsic hepatic pathology linked to TRIM37 dysfunction. The national cohort liver study by Sivunen et al. (2022), which included 21 biopsied patients, 17 patients assessed prior to pericardiectomy, and 36 outpatients in a cross-sectional evaluation, found sinusoidal dilatation, steatosis, and fibrosis as the main histological findings, with progression to cirrhosis in some patients [47]. In the biopsy group, serum levels of GGT, AST, ALT, and the AST-to-platelet ratio index were marginally elevated, with the proportion of affected patients ranging from 20% to 66% depending on the parameter. Similarly, in the pre-pericardiectomy group, these markers were elevated, with the proportion of affected patients ranging from 12% to 69% depending on the parameter. Transient elastography was elevated in 50% of patients with signs of heart failure. Importantly, GGT and total bilirubin showed significant age-related worsening in the outpatient cohort, suggesting worsening cholestatic features with age. Galactose half-life correlated significantly with transient elastography values (r = 0.48, *p* = 0.033) and with other markers of hepatic dysfunction including ALT, GGT, and APRI. Management of liver disease is primarily directed at treating the underlying cardiac disease. Limited pre- and post-pericardiectomy data from Sivunen et al. (2022) showed only marginal changes in liver transaminases after surgery, suggesting that hepatic improvement following pericardiectomy may be modest [47]. Patients with established liver disease may benefit from periodic assessment with liver function tests and non-invasive fibrosis markers. Advanced liver disease may complicate consideration of major cardiac interventions, including transplantation [28,47].

### 5.10. Tumour Surveillance and Management

MUL carries an increased risk of both benign and malignant tumours throughout life. In the national tumour cohort by Karlberg et al. (2009), 210 tumorous lesions were identified in 66 of 89 Finnish patients (74%). Fifteen malignancies were recorded in 13 patients (15%), comprising five Wilms’ tumours, two renal papillary carcinomas, three thyroid carcinomas, two gynaecological cancers, one gastrointestinal carcinoid, one case of neuropituitary Langerhans cell histiocytosis, and one case of acute lymphoblastic leukaemia [9]. Benign lesions included hepatic peliosis, cysts, adrenal and parathyroid adenomas, thyroid goitre, pancreatic cystadenoma, renal angiomyolipoma, ovarian fibrothecomas, and phaeochromocytoma. The most consistent hepatic finding was peliosis, likely reflecting a combination of elevated hepatic venous pressure and primary vascular dysregulation. Gynaecological tumours, particularly ovarian fibrothecomas, are a specific risk in postpubertal women with MUL. They may be bilateral or multifocal and have been linked to TRIM37 downregulation in ovarian stromal tissue [10]. Wilms’ tumour risk is mainly in childhood, as is typical for this malignancy. A pragmatic surveillance approach may include annual abdominal and pelvic ultrasonography throughout childhood to detect Wilms’ tumour and other renal or intra-abdominal lesions early, consistent with the practice of the Finnish national centre as described by Karlberg et al. (2009), where routine clinical follow-up included periodic abdominal ultrasonography [9]. In postpubertal women, regular gynaecological assessment (including pelvic ultrasonography) may be warranted given the ovarian tumour risk. Thyroid assessment may also be considered in adults given the described occurrence of thyroid malignancies, although no formal surveillance protocol for thyroid disease has been defined in the published MUL cohorts. Any new mass or unexplained systemic symptom warrants imaging and a low threshold for biopsy. The multidisciplinary nature of tumour management in MUL, particularly when cardiac or hepatic co-morbidities complicate anaesthesia and surgery, requires careful pre-treatment planning, as illustrated by the case of Upasana et al. (2022) in which doxorubicin was omitted and replaced with cyclophosphamide because of pre-existing right ventricular dysfunction [40].

### 5.11. Skeletal, Ophthalmological, and Pulmonary Care

In the cross-sectional radiographic study by Karlberg et al. (2025) of 33 Finnish patients aged 4.5–48 years, skeletal abnormalities were present in all participants. Fibrous dysplasia was present in 58% and a history of fractures in 52% [8]. The authors discuss multiple likely contributors to fracture risk: systemic factors such as hypogonadism, type 2 diabetes, feeding difficulties and malnutrition in early childhood, and low muscle tone, as well as structural bone changes including slender diaphyses, metaphyseal overmodelling, and bowing of the long bones. Given the high fracture burden and the reported lower-limb asymmetry in some patients, follow-up should include attention to fractures and limb asymmetry. Karlberg et al. note that bisphosphonate treatment may be indicated for some patients, however, no formal studies of bisphosphonate efficacy in MUL have been reported, and extrapolation from experience with fibrous dysplasia in other conditions should be made with caution. Karlberg et al. recommend calcium and vitamin D supplementation for MUL patients [8]. Given that characteristic yellowish retinal dots constitute a major diagnostic criterion [2], fundoscopic examination forms part of the diagnostic evaluation. These mid-peripheral dots were present in 79% of the Finnish diagnostic cohort [2]. Additional findings include retinal hypopigmentation with pigment dispersion and clustering [1,2] and strabismus, which was reported in some case series [24,58]. Visual acuity is generally preserved. In the review by Lapunzina et al. (1995), optic discs, maculae, visual acuity, and ERG were normal in reported patients [24]. This is consistent with the histopathological study of Tarkkanen et al. (1982), which demonstrated a well-differentiated retina at the posterior pole despite focal choroidal hypoplasia in the midperiphery [58]. Published case series have not described progression to clinically significant visual loss, although strabismus, when present, may warrant ophthalmological management. Pulmonary function in MUL is characterised by a restrictive pattern that appears to reflect restriction by an abnormally small, bell-shaped thoracic cage with thin ribs rather than intrinsic parenchymal disease, although cardiac dysfunction and pleural adhesions may also contribute. In the pulmonary cohort of Sivunen et al. (2020), total lung capacity and forced vital capacity were markedly reduced (51–63% of predicted), and FEV1 was likewise reduced (47–57%) [52]. Diffusing capacity (DLCO) was decreased (60–67% of predicted), but DLCO corrected for alveolar volume (DLCO/VA) was increased (130–148%), indicating preserved alveolar function despite the restrictive pattern. Bronchodilation tests were performed in 32 of 33 patients: only one met criteria for active asthma (FEV1 increase ≥12% and ≥200 mL), despite 4 patients (12%) using inhaled corticosteroids at the time of the study. The authors concluded that airflow limitation does not appear to be a typical feature in MUL and that volume restriction of the thoracic cage, rather than obstructive airway disease, is the dominant pulmonary pathophysiology. These findings raise the question of whether asthma diagnoses in MUL patients warrant reassessment, as symptoms attributed to obstructive disease may instead reflect the restrictive physiology of the small thorax.

### 5.12. Renal Surveillance

Renal anomalies, cystic lesions, and renal tumours have been reported in MUL. In the retrospective renal cohort of Sivunen et al. (2017) of 101 Finnish patients, structural anomalies of the kidneys and urinary tract were found in 13%, renal tumours in 14%, and macroscopic cystic lesions in 43% of the 81 patients examined by ultrasound [51]. Overall glomerular and tubular function was preserved in the cross-sectional subgroup of 36 patients evaluated clinically. However, moderate hypertension was common in those aged 16 years or older (42%), and only two patients had GFR below 60 mL/min/1.73 m^2^. Marginal elevation of plasma cystatin C was found in 10 of 27 evaluated patients (37%) and mild proteinuria in 7 of 26 (27%). The authors concluded that TRIM37 defects lead to an increased risk for renal anomalies, tumours, and cystic renal lesions, but not to progressive deterioration of renal function. At the Helsinki referral centre, abdominal ultrasonography was performed every 1–3 years in older patients and every 3–6 months in young children (under 6 years) to monitor for Wilms tumour growth. This surveillance protocol would also detect renal cysts when present. Given the high prevalence of hypertension in adult MUL patients, blood pressure monitoring and periodic assessment of renal function are reasonable considerations for long-term care.

### 5.13. Immune Surveillance and Infection Management

The immunological consequences of TRIM37 deficiency and the possible mechanisms, including a primary TRIM37–Bcl6-dependent immune defect and secondary PLE, are discussed in the Genetics and Molecular Pathogenesis and Clinical Features sections above. From a practical standpoint, recurrent respiratory tract infections in infancy are well documented [2], but the relative contributions of immune dysfunction, restrictive pulmonary physiology, feeding difficulties with aspiration risk, and muscular hypotonia to this susceptibility remain unclear. In the context of severe right-sided heart failure, PLE may develop and contribute secondarily to lymphopenia, hypoalbuminaemia, and impaired antibody levels. Distinguishing primary from secondary immune dysfunction is clinically important, as management differs. In reported MUL cases with suspected immune dysfunction, the immunological work-up has included measurement of immunoglobulin levels, lymphocyte subsets, and functional lymphocyte studies. Immunoglobulin replacement therapy may be considered on an individual basis after immunology evaluation and assessment of possible secondary protein loss, as Gazzin et al. (2023) observed that cessation of IVIG did not coincide with an increased infection rate and hypothesised that the immune deficiency in MUL may be substantially driven by hypercatabolic losses from PLE secondary to heart failure, although a contribution from intrinsic TRIM37-related defects cannot be excluded [17,18,50].

### 5.14. Prenatal Considerations

MUL may be worth considering in cases of unexplained prenatal growth restriction with additional anomalies. Tikanoja and Taipale (2004) reported a single case in which a fetus showed severe growth restriction, a nuchal fold of 7 mm, and progressive ascites from 28 weeks [59]. The karyotype was normal, and MUL was confirmed postnatally by identification of a homozygous TRIM37 deletion. Severe cardiac diastolic dysfunction was demonstrated by catheterisation (supraventricular tachycardia due to WPW syndrome was also noted, though the authors stated that WPW is not a known feature of MUL). The authors concluded that nuchal fold thickening and foetal ascites in this setting may be early signs of the diastolic dysfunction characteristic of MUL. Based on this single observation, molecular testing including TRIM37 may be considered when this constellation of findings is encountered and more common causes have been excluded. MUL follows autosomal recessive inheritance [1,2]. For families with a confirmed molecular diagnosis, pre-implantation genetic testing or prenatal diagnosis by chorionic villus sampling or amniocentesis may be considered, given the severity of the condition and the 25% recurrence risk. This recommendation reflects standard genetic counselling practice rather than MUL-specific published evidence.

### 5.15. Prognosis and Long-Term Outcomes

Long-term prognosis in MUL varies considerably and is mainly determined by the severity of cardiac involvement. In the national Finnish cohort of 49 patients followed by Lipsanen-Nyman et al. (2003) for up to 25 years, 22% died of cardiac causes and 10% of non-cardiac causes [3]. Cardiac deaths showed a bimodal pattern: significant early childhood mortality was followed by no deaths between ages 10 and 20, after which cardiac deaths began to occur again (median age at cardiac death 6.8 years, range 1.4–48). Importantly, however, 49% of patients (24/49) remained free of any symptomatic heart disease throughout follow-up. Long-term survival has been documented both with and without pericardiectomy: Cordova Sanchez et al. (2022) describe a patient who had mild diastolic dysfunction that remained stable for approximately 44 years after pericardiectomy at age 12, while maintaining education, employment, and community activity [55], while Lipsanen-Nyman et al. reported an unoperated 70-year-old woman doing well [3]. Beyond cardiac disease, several other factors contribute to long-term morbidity and influence prognosis. Malignancies were documented in 15% of the national tumour cohort of 89 patients (as detailed in the Tumour Surveillance section above). Renal and thyroid malignancies featured prominently, and Wilms’ tumour occurred in 5 of 89 patients (5.6%), with a mean age at diagnosis of 2.5 years [9]. Progressive hepatic disease is characterised by sinusoidal dilatation, steatosis, and fibrosis, with individual progression to cirrhosis, which Sivunen et al. (2022) found to be common in patients over 40 years of age [47]. The authors noted that while the changes are consistent with congestive hepatopathy, a TRIM37-independent contribution to liver pathology cannot be excluded. Advanced cirrhosis may limit candidacy for cardiac transplantation, although this has not been specifically studied in MUL. Metabolic syndrome (present in 70% of adults) and type 2 diabetes (in approximately half) are common in MUL [5]. These metabolic complications may worsen the cardiovascular disease. Skeletal dysplasia was present in all examined patients and fractures were reported in over half, predominantly low-energy injuries with occasionally impaired healing [8]. Gonadal failure in both sexes has lifelong endocrine and reproductive consequences [7,8,29]. Psychomotor development in infancy is normal or mildly delayed in a great majority of patients. Mild delays in motor development and speech are noted in approximately one third in infancy [2]. Individual case reports suggest that functional independence and engagement in education and employment are achievable [55].

In summary, prognosis is most favourable in patients who receive early diagnosis and cardiac intervention, undergo regular surveillance for oncological, hepatic, metabolic, and endocrine complications, and have access to multidisciplinary care throughout their lives. A single life expectancy estimate cannot be derived from the available data, which come from a founder population followed over a period when diagnostic and treatment standards were still evolving. Long-term outcomes are highly variable and largely determined by the severity and progression of cardiac involvement, as well as the burden of associated systemic complications. Improvements in diagnostic awareness and advances in supportive management have likely contributed to better survival in more recent cohorts. Lifelong follow-up remains essential, as new complications may emerge with age, particularly in adulthood. Further multicentre studies and international registries are needed to better define the natural history and optimize management strategies for this rare disorder.

Several important limitations and open questions remain. Most available clinical data and surveillance proposals are derived from Finnish cohorts, which represent the largest and best-characterised patient population but may not fully reflect the genetic and phenotypic diversity of MUL worldwide. From a translational perspective, experimental data suggest that TRIM37-deficient cells may be sensitive to centrosome loss, raising the question of whether PLK4 inhibition could have therapeutic relevance; however, this remains speculative and has not been evaluated in MUL patients. In addition, although TRIM37 has been implicated in epigenetic regulation, including H2A K119 mono-ubiquitination, the role of epigenetic mechanisms in the clinical phenotype of Mulibrey nanism is currently unknown. These issues highlight the need for further mechanistic and clinical studies.

## 6. Conclusions

Mulibrey nanism is a complex multisystem disorder with highly variable clinical expression and significant morbidity driven primarily by progressive cardiac disease. Advances in the understanding of TRIM37 function have provided important insights into disease mechanisms, although many aspects of its pathophysiology remain incompletely understood. The current evidence base is largely derived from relatively small cohorts, predominantly from the Finnish population, which may limit the generalisability of proposed surveillance strategies. Further research is needed to better define optimal management approaches and to explore emerging molecular mechanisms, including their potential therapeutic implications. International collaboration and larger multicentre studies will be essential to improve evidence-based care and long-term outcomes in this rare disorder.

## Figures and Tables

**Table 1:** Cumulative global case counts as estimated/reported by authors across key MUL publications. Finnish cases are shown in parentheses where reported. Approximate values (~) reflect the wording used in the original publications.

Cumulative Reported Cases (Global)	Source [Ref.]
~100 (80 Finnish)	Lipsanen-Nyman et al., 2003 [3]
~110 (85 Finnish)	Karlberg et al., 2004 [2]
115	Hämäläinen et al., 2006 [19]
~130 (88 Finnish)	Karlberg et al., 2007 [20]
135	Brigant et al., 2019 [21]
~150 (~110 Finnish)	Karlberg et al., 2025 [8]

**Table 2:** Published TRIM37 mutations associated with MUL. Reference sequence: NM_015294.2 (NM_015294.3 in Zodanu 2026 [39]). The c.80T>A (p.Leu27Gln) variant reported in the Indian population [40]. Some original publications use alternative TRIM37 reference transcripts (NM_001005207.2/.3 in Jobic 2017 [41], Mozzillo 2016 [38]; NM_001320987.3 in Gazzin 2023 [17]).

#	Mutation (cDNA)	Protein Change	Type	Exon/ Intron	Population	Reference(s)
1	c.493-2A>G	Aberrant splicing; protein truncated at ~174 aa	Splice-site	Intron 6	Finnish (Fin-major)	[31]
2	c.2212delG	Frameshift	Deletion	Exon 19	Finnish (Fin-minor)	[31]
3	c.80T>A	p.Leu27Gln	Missense	Exon 2	Indian	[40]
4	c.181C>T	p.Arg61*	Nonsense	Exon 4	French	[41]
5	c.227T>C	p.Leu76Pro	Missense	Exon 4	Finnish	[12]
6	c.326G>C	p.Cys109Ser	Missense	Exon 5	Australian	[19]
7	c.370-1G>A	Aberrant splicing	Splice-site	Intron 5	Iranian (Kurdish)	[42]
8	c.398C>T	p.Ala133Val	Missense	Exon 6	Turkish	[25]
9	c.685_809del	p.Leu229*	Large deletion	Exon 9	French	[41]
10	c.745C>T	p.Gln249*	Nonsense	Exon 9	Canadian	[43]
11	c.855-1G>A	Aberrant splicing	Splice-site	Intron 9	Turkish	[44]
12	c.838_842delACTTT	Frameshift	Deletion	Exon 10	Czech	[31]
13	c.860G>A	p.Glu271_Ser287del (in-frame exon 10 skipping)	Splice-site	Exon 10	Australian	[19]
14	c.874_877delAGAG	p.Arg292GlnfsX18	Deletion	Exon 11	Japanese	[45]
15	c.965G>T	p.Gly322Val	Missense	Exon 12	Canadian	[43]
16	c.1016C>G	p.Ser339Cys	Missense	Exon 12	Japanese	[45]
17	c.1020delC	p.Tyr341Ilefs*16	Deletion	Exon 12	Brazilian	[46]
18	c.1037_1040dupAGAT	p.Met347fs*7	Duplication	Exon 13	Canadian	[43]
19	c.1166A>G	Truncating (mechanism not specified)	Nonsense	Exon 13	Finnish	[47]
20	c.1233delA	Frameshift	Deletion	Exon 14	German	[48]
21	c.1314+507_1668-207del	Large intronic/exonic deletion	Large deletion	Introns 14 and 16	Sicilian	[43]
22	c.1315_1667del	p.Arg439Valfs*5	Large deletion	Exon 15-16	French	[41]
23	c.1336C>T	p.Arg446Ter	Nonsense	Exon 15	Indian	[40]
24	c.1346dupA	Frameshift	Duplication	Exon 15	American	[31]
25	c.1411C>T	p.Arg471*	Nonsense	Exon 15	Canadian/Tunisian	[43]
26	c.1894_1895delGA	p.Glu632fs	Deletion	Exon 18	Turkish	[25,49]
27	c.1949-12A>G	Aberrant splicing	Splice-site	Intron 18	Italian	[38,50]
28	c.2056C>T	p.Arg686*	Nonsense	Exon 19	Saudi Arabian	[43]
29	c.2357dupA	p.Asp786GlufsTer7	Duplication	Exon 21	Italian	[17]
30	del 17q22 (~143 kb)	Loss of TRIM37 gene product	Genomic deletion	17q22	Italian/other	[38,50]
31	del 17q22 (~60 kb, ex1-16)	Partial TRIM37 loss	Genomic deletion	17q22 (exons 1-16)	Italian	[17]
32	c.199C>T	p.Arg67Cys	Missense	Exon 4	Egyptian	[39]
33	c.2041C>G	p.Gln681Glu	Missense	Exon 19	Egyptian	[39]

## Data Availability

No new data were generated.

## Abbreviations

ALT, alanine aminotransferase; APRI, AST-to-platelet ratio index; AST, aspartate aminotransferase; BCL6, B-cell lymphoma 6; CHF, congestive heart failure; CTGF, connective tissue growth factor; CXCR5, C-X-C chemokine receptor type 5; DLCO, diffusing capacity of the lungs for carbon monoxide; DLCO/VA, DLCO corrected for alveolar volume; ECM, extracellular matrix; EMT, epithelial–mesenchymal transition; ERG, electroretinography; FEV1, forced expiratory volume in 1 s; FSH, follicle-stimulating hormone; FVC, forced vital capacity; GFR, glomerular filtration rate; GGT, gamma-glutamyltransferase; GH, growth hormone; IVIG, intravenous immunoglobulin; LH, luteinising hormone; MATH, meprin and TRAF-C homology; MRI, magnetic resonance imaging; MTOR, mechanistic target of rapamycin; mTORC1, mechanistic target of rapamycin complex 1; MUL, Mulibrey nanism; OMIM, Online Mendelian Inheritance in Man; PEX5, peroxin 5; PLE, protein-losing enteropathy; RBCC, RING-B-box-coiled-coil; RRAGB, Ras-related GTP-binding protein B; SDS, standard deviation score; SPARC, Secreted Protein Acidic and Rich in Cysteine; TFEB, transcription factor EB; TFH, T follicular helper; TGF-β, transforming growth factor-beta; TLC, total lung capacity; TRAF, tumour necrosis factor receptor-associated factor; TRIM37, tripartite motif protein 37; WPW, Wolff–Parkinson–White; α-SMA, alpha smooth muscle actin.
